# Association between the central sensitization inventory score and health-related quality of life in community-dwelling middle-aged and older adults

**DOI:** 10.1371/journal.pone.0335923

**Published:** 2025-10-30

**Authors:** Naoki Segi, Hiroaki Nakashima, Ryotaro Oishi, Sadayuki Ito, Jun Ouchida, Ippei Yamauchi, Yasuhiro Nagatani, Taisuke Seki, Yasuhiko Takegami, Shinya Ishizuka, Yukiharu Hasegawa, Shiro Imagama

**Affiliations:** 1 Department of Orthopaedic Surgery, Nagoya University Graduate School of Medicine, Nagoya, Japan; 2 Department of Orthopedic Surgery, Japanese Red Cross Aichi Medical Center Nagoya Daiichi Hospital, Nagoya, Aichi, Japan; 3 Department of Orthopedic Surgery, Aichi Medical University Medical Center, Okazaki, Aichi, Japan; 4 Biomedical Imaging and Informatics, Department of Integrated Health Sciences, Nagoya University Graduate School of Medicine, Nagoya, Japan; 5 Department of Rehabilitation, Kansai University of Welfare Sciences, Kashiwara, Osaka, Japan; Mie University Graduate School of Medicine, JAPAN

## Abstract

**Background:**

Central sensitization is an important factor associated with impaired health-related quality of life in patients with musculoskeletal disorders and community-dwelling older adults. However, health-related quality-of-life domains strongly associated with central sensitization in the general population remain unclear. This study aimed to examine the association between the Central Sensitization Inventory Part A scores and health-related quality of life using community health checkup data.

**Methods:**

A total of 419 middle-aged and older adults (mean age, 64.4 ± 11.2 years; 59.4% female) were included. Participants completed a questionnaire survey on pain, including visual analogue scales (VASs) for lower-back and knee pain, and the Central Sensitization Inventory Part A. Additionally, participants completed the Short-Form 36-Item Health Survey, and three component-summary scores and eight subscales were calculated. Additionally, participants completed the 5-level EuroQol 5 dimensions, and health-state utility values were calculated. The correlation between the Central Sensitization Inventory Part A scores and these health-related quality-of-life measures was investigated.

**Results:**

Central Sensitization Inventory Part A score ≥40 was observed in 2.6% participants. Significant moderate negative correlations were observed between the Central Sensitization Inventory Part A scores and EuroQol 5 dimensions health-state utility values (**r* *= −0.631, **P* *< 0.001), Short-Form 36 mental-component summary (**r* *= −0.550, **P* *< 0.001), body pain (**r* *= −0.556, **P* *< 0.001), general health (**r* *= −0.556, **P* *< 0.001), vitality (**r* *= −0.610, **P* *< 0.001), and mental health (**r* *= −0.556, **P* *< 0.001). Similar results were obtained for participants with Central Sensitization Inventory Part A scores <30.

**Conclusions:**

In community-dwelling middle-aged and older adults, Central Sensitization Inventory Part A scores were negatively correlated with health-related quality-of-life scores, even in participants with Central Sensitization Inventory Part A scores <30.

## Introduction

Central sensitization (CS) [1] is an increasingly recognized key factor in the pathophysiology of various painful conditions, including musculoskeletal disorders, migraine, and chronic pain [2]. CS is closely associated with nociplastic pain [3] and is characterized by increased responsiveness of pain neurons in the central nervous system to normal or subthreshold afferent inputs [4]. The pathophysiological mechanisms of CS include the activation of peripheral pain-sensitive C-fibers by various chemical substances, such as substance P, serotonin, and bradykinin, following painful stimuli or inflammatory states [5]. The severity of CS is generally evaluated using the CS Inventory (CSI) [6–8]. The CSI consists of Part A (CSI-A), which includes 25 questions related to CS, and Part B, which investigates the presence of any of the eight CS-related diagnoses.

With improved understanding of CS, targeted pharmacological interventions and cognitive behavioral therapy are being developed and refined to improve treatment outcomes [9]. In patients with musculoskeletal disorders, CS is associated with impaired health-related quality of life (HRQOL) [10–12], suggesting that addressing CS through appropriate screening and management strategies may improve patients’ HRQOL. Additionally, CS negatively impacts HRQOL measured using EuroQol 5 dimensions (EQ5D) utility values in community-dwelling older adults [13].

Considering that several community-dwelling older adults experience chronic pain [14] and the expected global increase in the older-adult population, investigating the relationship between CS, which is closely associated with chronic pain, and HRQOL in community residents is important. However, the HRQOL domains strongly associated with CS or its precursors in the general population remain unclear. Furthermore, although the prevalence of CS in the general population at a cutoff CSI-A score ≥40 is approximately 4% [15,16], the association between CSI-A and HRQOL component scores in community residents remains unclear, regardless of the cutoff. Understanding the burden of pain and CS-related symptoms assessed using CSI among community residents and their association with HRQOL is an important topic in public health. Therefore, this study aimed to examine the association between CSI-A scores and HRQOL using cross-sectional data from community health examinations.

## Materials and methods

### Study population

The study included middle-aged and older volunteers who participated in the Yakumo study [17–19], which consists of annual community health checkups since 1982, including internal medicine, orthopedic, and psychiatric evaluations. An announcement outlining the aims of the health screening program is mailed annually to residents of Yakumo town aged ≥40 years, and the annual response rate is approximately 12%. Therefore, the study population mainly consisted of residents who were physically active and independent regarding activities of daily living.

**Fig 1:**
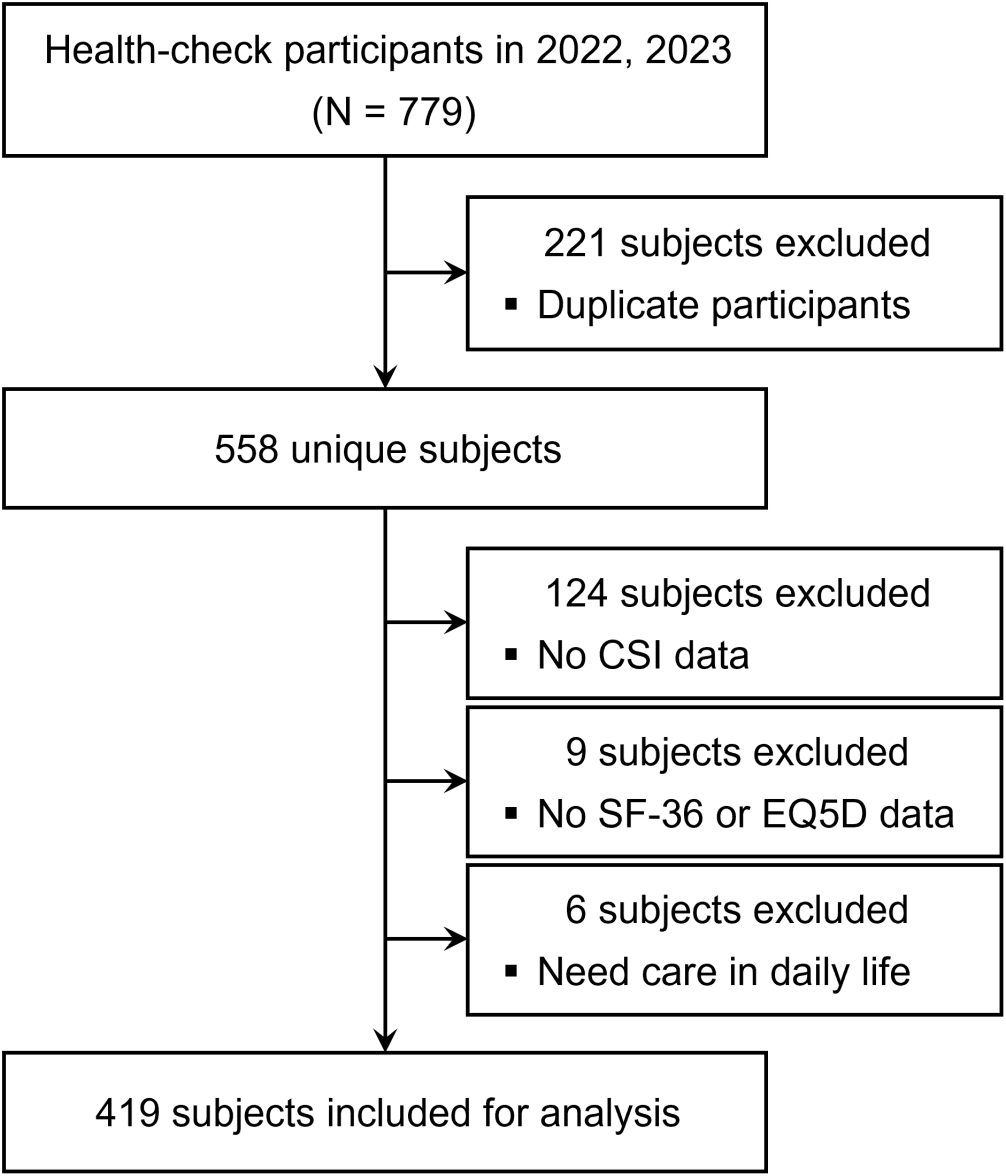
Participant selection flowchart. CSI, Central Sensitization Inventory; SF-36, 36-Item Short-Form Health Survey; EQ5D, EuroQol 5 dimensions.

A total of 779 residents who participated in the Hokkaido Yakumo Town Resident Health Screening in 2022 and 2023 were designated as the initial cohort. Of these, we excluded 124, 9, and 6 participants who did not respond to CSI, had missing SF-36 and EQ-5D data, and required care for daily living, respectively. For the 221 individuals who participated in both 2022 and 2023, data from 2022 alone were used, and duplicates were excluded. Finally, data from 419 participants (75.1%) were included in the analysis (Fig 1).

The study protocol adhered to the principles of the Declaration of Helsinki and was approved by the Human Research Ethics Committee and Institutional Review Board of Nagoya University (No. 2022−0328). Written informed consent was obtained from all participants. The dataset was accessed on September 1, 2024, for the purposes of this study. No information that could identify individual participants was accessed during or after data collection.

### Variables

Participants completed a questionnaire survey on pain, including VASs for lower-back and knee pain, and the CSI. Additionally, participants completed the Medical Outcome Study Short-Form 36-Item Health Survey (SF-36, Japanese version 2.0), encompassing three component-summary scores based on standardized Japanese values — physical component summary, mental component summary (MCS), and role/social component summary— and eight subscales (physical functioning, role-physical [RP], body pain [BP], general health [GH] perception, vitality [VT], social functioning, role-emotional [RE], and mental health [MH]).

In addition, participants completed the 5-level EQ5D (EQ5D-5L), and health-state utility values (HSUVs) were calculated [20,21]. Furthermore, presence of the following comorbidities was evaluated: hypertension, malignant diseases (previous and current), diabetes, chronic kidney disease, angina or myocardial infarction, and stroke. Additionally, participants were asked about their exercise habits – specifically, whether they engaged in physical activity for ≥1–2 h per week.

### Statistical analysis

Data are presented as means ± standard deviations for continuous variables and as numbers and percentages for categorical variables. Statistical analyses were performed using R version 4.4.0 (http://www.R-project.org, 2024-04-24) with the tidyverse [22] and gtsummary [23] packages. Comparisons between groups were performed using the Wilcoxon rank sum test, Fisher’s exact test, and Pearson’s Chi-square test. Statistical significance was set at p < 0.05. We visualized the relationship between CSI-A scores and HRQOL indicators using a spline model based on a generalized additive model.

The association between the total CSI score and the EQ5D HSUV or SF-36 scores was evaluated using multivariable linear regression analysis. Using three levels of sequential adjustments, the following models were developed: 1) unadjusted model, 2) age- and sex-adjusted model (including age as a continuous variable and sex), and 3) fully adjusted model (including age; sex; comorbidities such as hypertension, malignant neoplasm, diabetes, chronic kidney disease, angina or myocardial infarction, and cerebral stroke; and exercise habit).

For sensitivity analysis, the same analysis was conducted using data from participants with CSI-A scores <30 (S1 Fig). Correlations between CSI-A scores and EQ5D, SF-36 summary scores and SF-36 subscale scores were examined.

## Results

**Fig 2:**
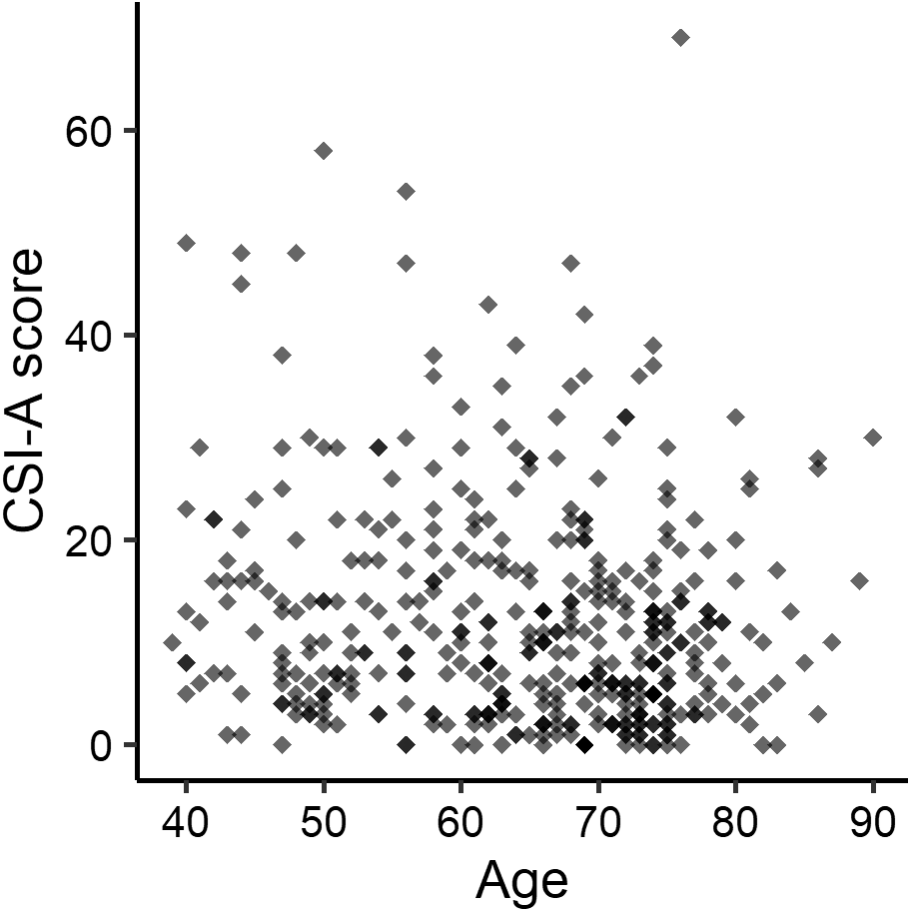
Distribution of total CSI-A scores according to age. CSI-A, Central Sensitization Inventory, Part A.

**Fig 3:**
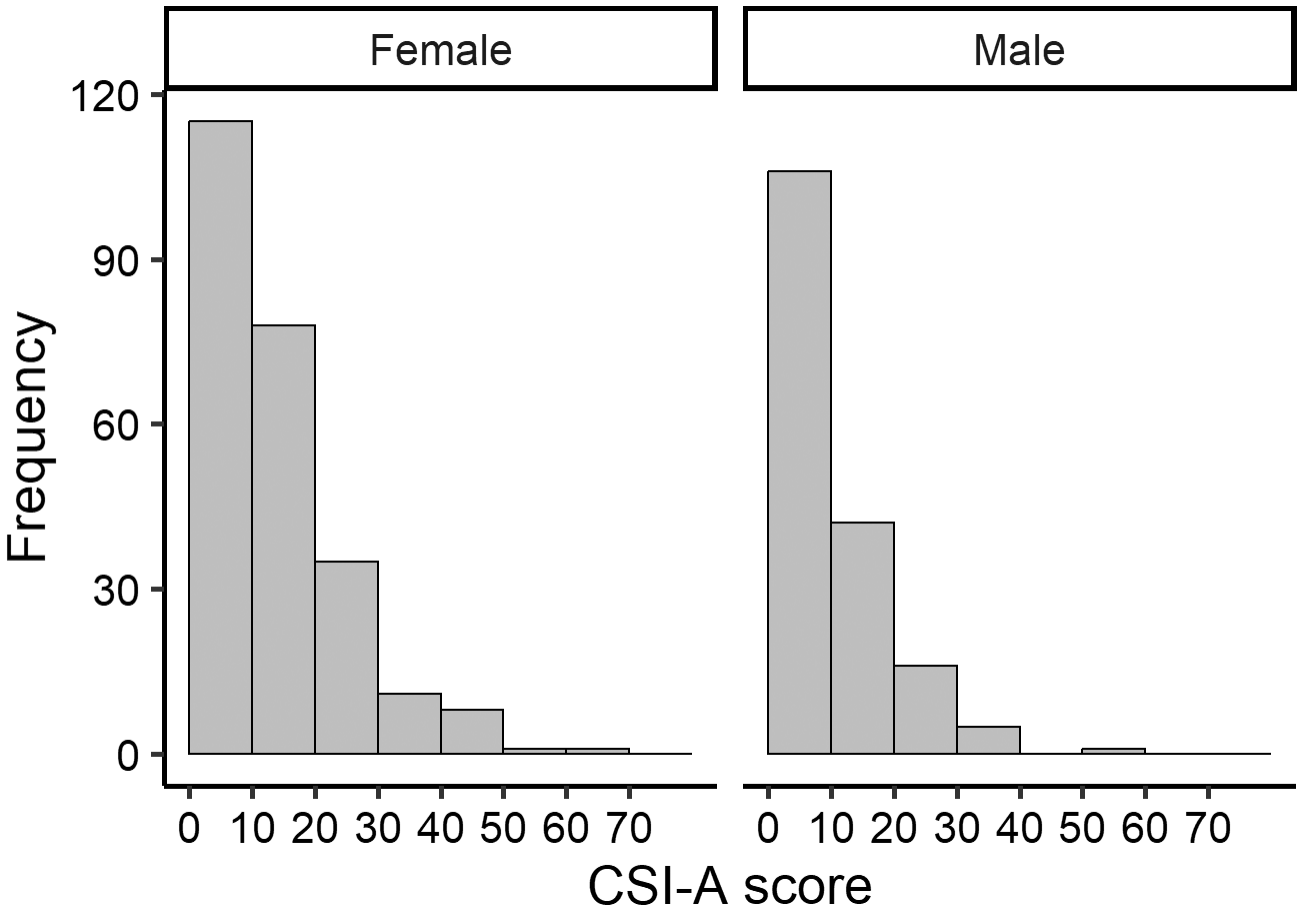
Distribution of total CSI-A scores according to sex. CSI-A, Central Sensitization Inventory, Part A.

The mean age of the participants was 64.4 ± 11.2 years (range, 39–90 years), and 59.4% of participants were female. The mean VAS scores for lower-back and knee pain were 13.3 mm and 10.9 mm, respectively (Table 1). The mean CSI-A score was 12.4 ± 10.9 points, and 11 participants (2.6%) had a CSI score ≥40 points. A small correlation was observed between CSI-A scores and age (**r* *= −0.130, Fig 2). The CSI-A score histograms for males and females showed similar shapes (Fig 3).

**Table 1:** Analytic cohort characteristics.

	N = 419
**Age, years**	64.4 ± 11.2
min, max	39.0, 90.0
**Sex, female**	249 (59.4%)
**Comorbidities**	
Hypertension	145 (34.6%)
Malignant neoplasm	73 (17.4%)
Diabetes	31 (7.4%)
Chronic kidney disease	26 (6.2%)
Angina or myocardial infarction	19 (4.5%)
Cerebral stroke	10 (2.4%)
Exercise habit*	168 (40.1%)
**Pain, VAS (mm)**	
Lower-back pain	13.3 ± 21.0
Knee pain	10.9 ± 19.4
**CSI-A score**	12.4 ± 10.9
CSI-A ≥ 40 (moderate or higher)	11 (2.6%)
**EQ5D health state utility value**	0.888 ± 0.125
**SF-36**	
Physical component summary	48.7 ± 11.0
Mental component summary	51.8 ± 9.6
Role component summary	49.5 ± 10.6
Physical functioning	86.7 ± 16.7
Role physical	88.0 ± 18.6
Bodily pain	70.3 ± 22.6
General health	69.3 ± 18.9
Vitality	61.3 ± 18.8
Social functioning	86.5 ± 20.0
Role emotional	88.7 ± 19.1
Mental health	74.7 ± 17.0

*Exercise ≥1–2 h per week.

VAS, visual analogue scale; CSI-A, Central Sensitization Inventory, Part A; EQ5D, EuroQol 5 dimensions; SF-36, 36-Item Short-Form Health Survey.

**Fig 4:**
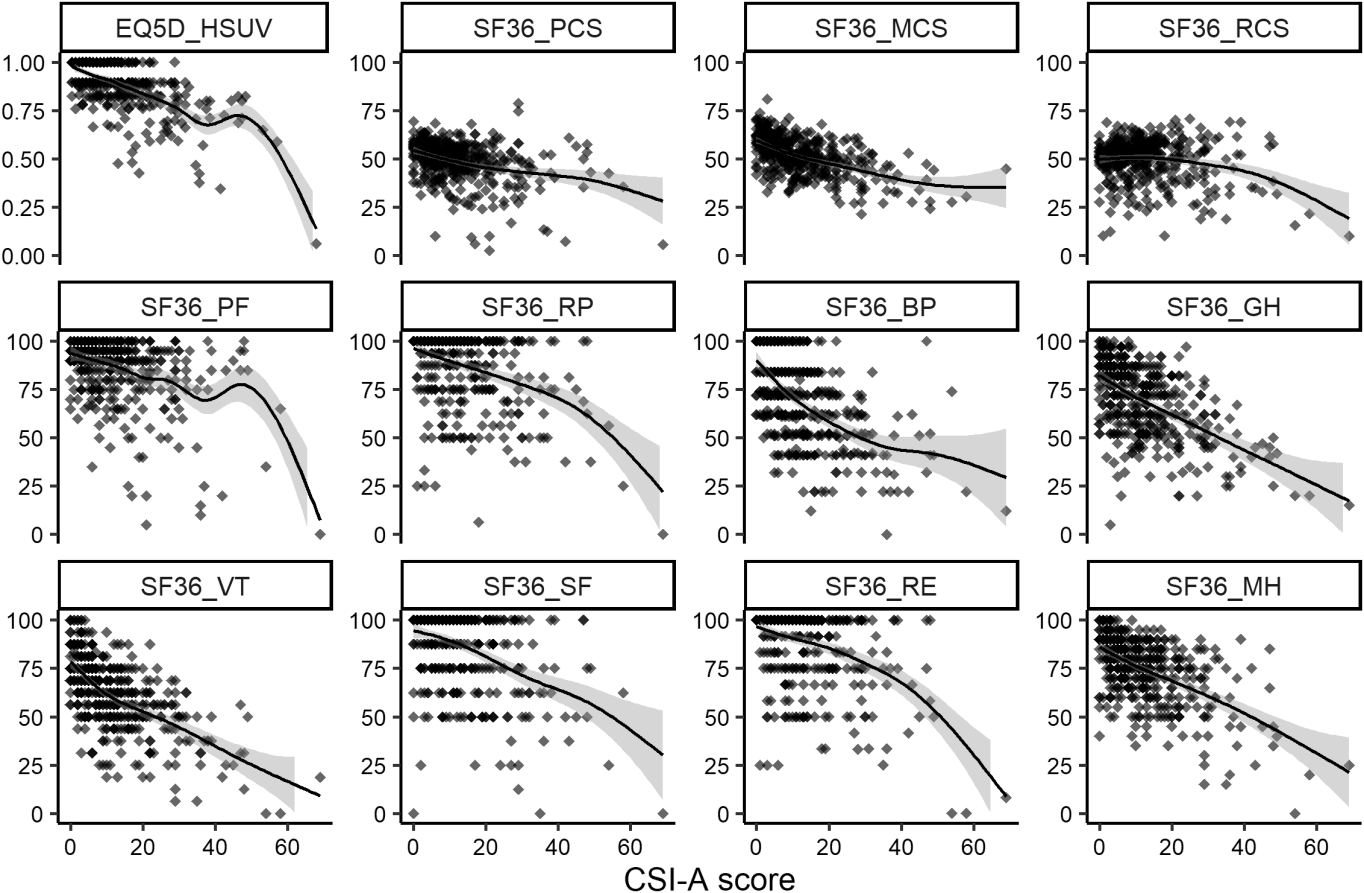
Correlation between CSI-A score and HRQOL indicators. CSI-A, Central Sensitization Inventory, Part A; HRQOL, health-related quality of life; EQ5D, EuroQol 5 dimensions; HSUV, health-state utility value; SF-36, 36-Item Short-Form Health Survey; PCS, physical component summary; MCS, mental component summary; RCS, role component summary PF, physical functioning; RP, role physical; BP, bodily pain; GH, general health; VT, vitality; SF, social functioning; RE, role emotional; MH, mental health.

CSI-A scores were significantly negatively correlated with EQ5D HSUV (**r* *= −0.631, **P* *< 0.001), MCS (**r* *= −0.550, **P* *< 0.001), BP (**r* *= −0.556, **P* *< 0.001), GH (**r* *= −0.556, **P* *< 0.001), VT (**r* *= −0.610, **P* *< 0.001), and MH (**r* *= −0.556, *P* < 0.001) with correlation coefficients <−0.5 (Table 2). Additionally, scatter plots with spline models suggested nonlinearity, with negative correlations with EQ5D HSUV, SF-36 PF, RP, and RE increasing at a CSI-A threshold of 40 points. In contrast, variables such as MCS, GH, VT, and MH showed relatively linear associations (Fig 4). Multivariable linear regression analysis revealed that CSI-A scores were independently and negatively correlated with EQ5D HSUV, SF-36 summary scores, and SF-36 subscale scores (Table 3).

**Table 2:** Correlation between CSI-A score and HRQOL indicators.

	Estimate	95% CI	*P* value
EQ5D HSUV	−0.631	−0.685, −0.569	<0.001
SF36 PCS	−0.370	−0.450, −0.285	<0.001
SF36 MCS	−0.550	−0.614, −0.480	<0.001
SF36 RCS	−0.193	−0.284, −0.099	<0.001
SF36 PF	−0.388	−0.467, −0.304	<0.001
SF36 RP	−0.413	−0.489, −0.330	<0.001
SF36 BP	−0.556	−0.618, −0.486	<0.001
SF36 GH	−0.556	−0.619, −0.486	<0.001
SF36 VT	−0.610	−0.667, −0.546	<0.001
SF36 SF	−0.444	−0.517, −0.363	<0.001
SF36 RE	−0.442	−0.516, −0.361	<0.001
SF36 MH	−0.556	−0.618, −0.486	<0.001

CSI-A, Central Sensitization Inventory, Part A; HRQOL, health-related quality of life; CI, confidence interval; EQ5D, EuroQol 5 dimensions; HSUV, health-state utility value; SF-36, 36-Item Short-Form Health Survey; PCS, physical component summary; MCS, mental component summary; RCS, role component summary PF, physical functioning; RP, role physical; BP, bodily pain; GH, general health; VT, vitality; SF, social functioning; RE, role emotional; MH, mental health.

**Table 3:** Multivariable linear regression analysis to determine the association of CSI-A score with HRQOL indicators.

	Unadjusted			Age- and sex-adjusted			Fully adjusted*		
	Beta	95% CI	*P* value	Beta	95% CI	*P* value	Beta	95% CI	*P* value
EQ5D HSUV	−0.01	−0.01, −0.01	<0.001	−0.01	−0.01, −0.01	<0.001	−0.01	−0.01, −0.01	<0.001
SF36 PCS	−0.37	−0.46, −0.28	<0.001	−0.42	−0.50, −0.33	<0.001	−0.37	−0.46, −0.29	<0.001
SF36 MCS	−0.48	−0.56, −0.41	<0.001	−0.47	−0.54, −0.41	<0.001	−0.47	−0.55, −0.40	<0.001
SF36 RCS	−0.19	−0.28, −0.10	<0.001	−0.21	−0.30, −0.11	<0.001	−0.21	−0.31, −0.12	<0.001
SF36 PF	−0.59	−0.73, −0.46	<0.001	−0.65	−0.78, −0.52	<0.001	−0.60	−0.73, −0.47	<0.001
SF36 RP	−0.70	−0.85, −0.55	<0.001	−0.77	−0.91, −0.62	<0.001	−0.73	−0.88, −0.58	<0.001
SF36 BP	−1.2	−1.3, −0.99	<0.001	−1.2	−1.4, −1.0	<0.001	−1.2	−1.3, −0.99	<0.001
SF36 GH	−0.96	−1.1, −0.83	<0.001	−1.0	−1.2, −0.88	<0.001	−0.96	−1.1, −0.82	<0.001
SF36 VT	−1.0	−1.2, −0.92	<0.001	−1.0	−1.2, −0.90	<0.001	−1.0	−1.2, −0.89	<0.001
SF36 SF	−0.81	−0.97, −0.65	<0.001	−0.82	−0.98, −0.66	<0.001	−0.81	−0.98, −0.64	<0.001
SF36 RE	−0.77	−0.93, −0.62	<0.001	−0.84	−0.99, −0.70	<0.001	−0.83	−0.98, −0.68	<0.001
SF36 MH	−0.86	−0.99, −0.74	<0.001	−0.88	−1.0, −0.75	<0.001	−0.88	−1.0, −0.75	<0.001

*The fully adjusted model includes age, sex, comorbidities (hypertension, malignant neoplasm, diabetes, chronic kidney disease, angina or myocardial infarction, and cerebral stroke), and exercise habit as covariates.

CSI-A, Central Sensitization Inventory, Part A; HRQOL, health-related quality of life; CI, confidence interval; EQ5D, EuroQol 5 dimensions; HSUV, health-state utility value; SF-36, 36-Item Short-Form Health Survey; PCS, physical component summary; MCS, mental component summary; RCS, role component summary PF, physical functioning; RP, role physical; BP, bodily pain; GH, general health; VT, vitality; SF, social functioning; RE, role emotional; MH, mental health.

The spline model is based on the generalized additive model.

Among participants with CSI-A scores <30, the correlation coefficients for each item with CSI-A were as follows: EQ5D HSUV (**r* *= −0.492, **P* *< 0.001), MCS (**r* *= −0.474, **P* *< 0.001), BP (**r* *= −0.506, **P* *< 0.001), GH (**r* *= −0.411, **P* *< 0.001), VT (**r* *= −0.508, **P* *< 0.001), MH (**r* *= −0.435, **P* *< 0.001) (S2 Fig, S1 Table). Multivariable linear regression analysis confirmed that CSI-A scores were independently and negatively correlated with all items except SF36 role/social component summary (S2 Table).

## Discussion

In this study, higher CSI-A scores were associated with poor HRQOL, particularly in the mental domain than in the physical domain. The association remained significant even after adjusting for confounding factors, demonstrating that CSI-A scores are associated with health perception among community residents, regardless of its severity. Similar results were obtained for participants with CSI-A scores < 30. However, CSI-A and MH assessments share overlapping or highly similar concepts in their measurement. Moreover, the study included participants with minimal pain burden.

This study not only reconfirmed the significant correlation between poor CSI-A scores and poor HRQOL among community-dwelling middle-aged and older adults but also clarified the details of this correlation. Few studies have investigated the association between CS and HRQOL without targeting specific disease cohorts. In a previous study involving 206 community-dwelling older adults (age ≥ 60 years), a significant negative correlation (*r*=−0.409, *P* < 0.001) was found between CS assessed using the CSI short version and EQ5D HSUV [13]. However, the HRQOL domains strongly associated with CS in community-dwelling individuals remained unclear. In contrast, this study identified HRQOL items strongly correlated with CS in community-dwelling individuals through an analysis of SF-36 subscales.

CSI-A scores were more frequently associated with HRQOL parameters in subscales reflecting MH (MCS, VT, MH) than in those reflecting physical aspects. As expected, exposure to chronic pain due to CS-related burden was associated with poor pain-domain (BP) and overall-health (GH, HSUV) scores. However, poor MCS, VT, and MH scores were also significantly associated with CSI-A scores. Notably, CS is not only associated with primary conditions such as anxiety, depression, and sleep disorders, but also with psychosocial factors such as fear, pessimistic thinking, and anxiety [15,24]. Thus, more than half of the variability in CSI scores in disease cohorts was attributed to psychological factors, and strong associations have been found between CSI and MH indicators [25,26]. Thus, whether these disorders cause CS, CS modifies the mental domain, or the variables interact in a feedback loop remains unclear. Nonetheless, the finding that higher CSI-A scores and MH issues are prevalent even among independent community-dwelling individuals is an important insight.

The association between CSI-A scores and HRQOL was relatively linear, particularly in terms of MCS, SF-36 VT, and MH (Figs 4 and S2). CSI was originally developed as a system for qualitatively diagnosing CS, with the threshold value generally set at 40 points [6]. Grading based on scores has been considered [8]; however, CSI is not primarily intended for quantitative evaluation. Nevertheless, the results of this study suggest that even low CSI-A scores are moderately negatively correlated with HRQOL. This correlation could have been better elucidated through screening of the general population than of a disease cohort. Furthermore, the EQ5D HSUV and several SF-36 subscales suggested non-linearity around a CSI-A score of 40 (Fig 4). This finding implies that the threshold of 40 points, established in disease cohorts, may also be relevant for community-dwelling populations.

Nonetheless, the results of this study may merely represent a spurious correlation, as MH issues inherent in the general population in modern society — such as subclinical mood and anxiety disorders [27] — are evaluated using similar questions in both the CSI and SF-36 scales. In MH assessments using the CSI-A and SF-36, measurement items overlap or include similar questions, potentially leading to partial linear variation between the CSI-A and SF-36 scales. Nevertheless, this study opens new possibilities for the use of CSI as a screening tool for community MH [25,26]. Interestingly, even in the group with CSI-A scores below the CS threshold, a negative correlation was observed between CSI-A scores and HRQOL. This finding is intriguing because it suggests that CSI-A scores can be used to estimate the MH burden even in participants who may not be initially diagnosed with CS. The CSI-A, which fits on a single sheet of paper and includes only 25 questions, is an ideal screening tool. However, because the CSI was not originally designed as an MH screening tool, caution is necessary when using it for this purpose [28], and further studies on this topic are warranted.

Compared to the association between the mental domain and CSI-A scores, the associations between domains interpreted as physical or social and CSI-A scores were weaker. However, the influence of CS on the physical condition or social situation of community residents cannot be ruled out [29]. Several SF-36 subscales exhibited potential non-linearity near the CSI-A cut-off of 40 (Fig 4). Although subscales with stronger correlations (e.g., MCS, VT, MH) displayed relatively linear associations, physical and social subscales appeared more susceptible to non-linear changes. However, because only a limited number of participants had CSI-A scores ≥40, the robustness of these findings remains uncertain, and additional studies with larger sample sizes are warranted to verify our results.

Although this study targeted a broad population comprising community-dwelling middle-aged and older adults, caution is warranted regarding its generalizability. In our country, the prevalence of CS, diagnosed based on a threshold CSI-A score of 40, in the general population is estimated to be approximately 4% [15,16]. Additionally, the prevalence of mental disorders is higher in high-latitude regions [30]. However, only 2.6% of participants had a CSI-A score ≥40, indicating that the prevalence of CS in the study cohort was lower than that in the general population. This could be attributed to selection bias, as participants’ response rate was relatively low (approximately 12%), potentially leading to the selection of a healthier and more active cohort. Other possible explanations could include better physical health, a habit of regular exercise, strong community ties, or longer health span and life expectancy. For example, the prevalence of hypertension and diabetes mellitus among participants was lower than the national averages (47.7% and 14.6%, respectively) [31]. Furthermore, participants in the Yakumo study tended to maintain higher physical capabilities from the perspective of locomotive syndrome [32]. Additionally, the average exercise habit among participants was approximately 40%, which was higher than the 2023 national average (28.6% for women, 36.2% for men) [33], indicating a relatively larger proportion of active residents. Furthermore, compared to urban areas, rural communities often have stronger community ties, which positively influence subjective well-being [34]. Although the strength of community ties in this region has not been quantified, Yakumo Town is considered a rural area. These factors may have contributed to the potentially lower prevalence of mental disorders. In contrast, average life expectancy in Yakumo Town in 2020 was 87.2 years for women and 80.7 years for men, which is comparable to the national average (87.6 and 81.5 years, respectively) [33]. Similarly, the healthy life expectancy in Yakumo Town in 2019 was 66.5 years for women and 65.2 years for men, which is comparable to the national average (66.8 and 65.2 years, respectively) [35]. Therefore, the impact of these factors is likely to be limited.

This study had some limitations. First, the selection bias introduced by the inclusion of relatively healthy volunteers who participated in health checkups may have affected the generalizability of our findings. In this study, the average VAS score for pain was low, indicating that the analysis was based on a sample with a small pain burden. Second, because this was a cross-sectional study, causal relationships could not be determined. Moreover, we acknowledge the possibility of reverse causality in the observed associations between CSI-A scores and MH domains. Third, the Yakumo study mainly focused on the health status of the organs and musculoskeletal systems and QOL; therefore, data on participants’ educational level, socioeconomic status, or clinical MH diagnoses were not available. Thus, these factors, which should have been adjusted for, were not included in the model. Finally, single assessment tools, such as the CSI, may not fully elucidate the complexity of CS. CSI is a symptom-based, self-reported questionnaire designed to assess CS-related symptom burden and not to provide a definitive diagnosis of CS or measure its neurophysiological mechanisms. Therefore, overinterpreting CSI scores as direct evidence of CS must be avoided.

## Conclusion

Although CSI-A and MH assessments share measurement metrics, this cross-sectional study revealed that CSI-A scores were negatively correlated with HRQOL, particularly in the mental domain, in community-dwelling middle-aged and older adults. Furthermore, CSI-A scores were negatively correlated with HRQOL even below the generally accepted threshold.

## Supporting information

S1 Fig Age and total CSI-A score distribution in SCI-A score <30 cohort. CSI-A, Central Sensitization Inventory, Part A.
(PDF)

S2 Fig Correlation between CSI-A score and HRQOL indicators in SCI-A score <30 cohort. CSI-A, Central Sensitization Inventory, Part A; HRQOL, health-related quality of life; EQ5D, EuroQol 5 dimensions 5-level; HSUV, health state utility value; SF-36, 36-Item Short-Form Health Survey; PCS, physical component summary; MCS, mental component summary; RCS, role component summary PF, physical functioning; RP, role physical; BP, bodily pain; GH, general health; VT, vitality; SF, social functioning; RE, role emotional; MH, mental health. The spline model is based on the generalized additive model.
(PDF)

S1 Table Correlation between CSI-A score and HRQOL indicators in SCI-A score <30 cohort. CSI-A, Central Sensitization Inventory, Part A; HRQOL, health-related quality of life; CI, confidence interval; EQ5D, EuroQol 5 dimensions; HSUV, health-state utility value; SF-36, 36-Item Short-Form Health Survey; PCS, physical component summary; MCS, mental component summary; RCS, role component summary PF, physical functioning; RP, role physical; BP, bodily pain; GH, general health; VT, vitality; SF, social functioning; RE, role emotional; MH, mental health.
(PDF)

S2 Table Multivariable linear regression analysis to determine the association of CSI-A score with HRQOL indicators in SCI-A score <30 cohort. CSI, Central Sensitization Inventory; HRQOL, health-related quality of life; CI, confidence interval; EQ5D, EuroQol 5 dimensions; HSUV, health-state utility value; SF-36, 36-Item Short-Form Health Survey; PCS, physical component summary; MCS, mental component summary; RCS, role component summary PF, physical functioning; RP, role physical; BP, bodily pain; GH, general health; VT, vitality; SF, social functioning; RE, role emotional; MH, mental health. *The fully adjusted model includes age, sex, comorbidities (hypertension, malignant neoplasm, diabetes, chronic kidney disease, angina or myocardial infarction, and cerebral stroke), and exercise habit as covariates.
(PDF)
