# Supplementary material for: Association between the central sensitization inventory score and health-related quality of life in community-dwelling middle-aged and older adults
Source: PLoS One. 2025 Oct 30;20(10):e0335923. doi: 10.1371/journal.pone.0335923 (PMC12574846; doi:10.1371/journal.pone.0335923)
Supplement: S1 Fig — CSI-A, Central Sensitization Inventory, Part A. (PDF) [file pone.0335923.s001.pdf]

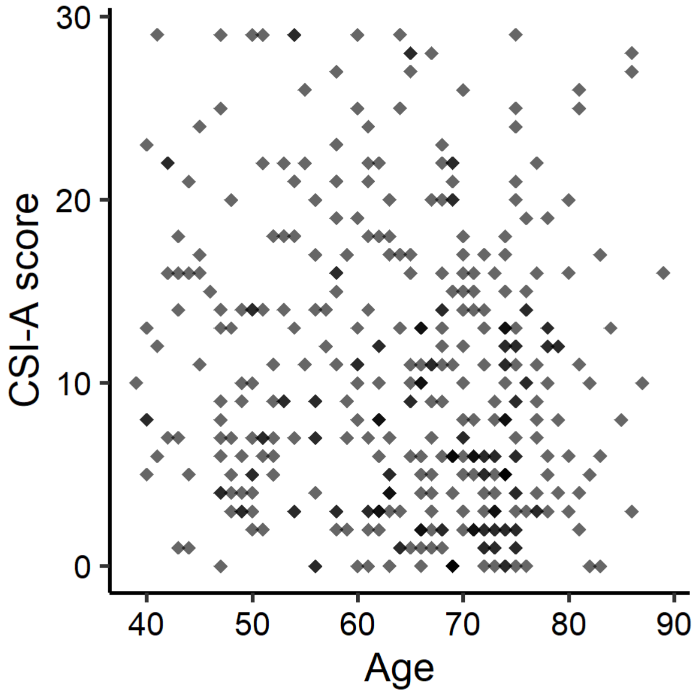

Supplemental Figure 1.
Age and total CSI-A score distribution in SCI-A score <30 cohort

CSI-A, Central Sensitization Inventory, Part A.
