# Supplementary material for: Association between the central sensitization inventory score and health-related quality of life in community-dwelling middle-aged and older adults
Source: PLoS One. 2025 Oct 30;20(10):e0335923. doi: 10.1371/journal.pone.0335923 (PMC12574846; doi:10.1371/journal.pone.0335923)
Supplement: S2 Fig — CSI-A, Central Sensitization Inventory, Part A; HRQOL, health-related quality of life; EQ5D, EuroQol 5 dimensions 5-level; HSUV, health state utility value; SF-36, 36-Item Short-Form Health Survey; PCS, physical component summary; MCS, mental component summary; RCS, role component summary PF, physical functioning; RP, role physical; BP, bodily pain; GH, general health; VT, vitality; SF, social functioning; RE, role emotional; MH, mental health. The spline model is based on the generalized additive model. (PDF) [file pone.0335923.s002.pdf]

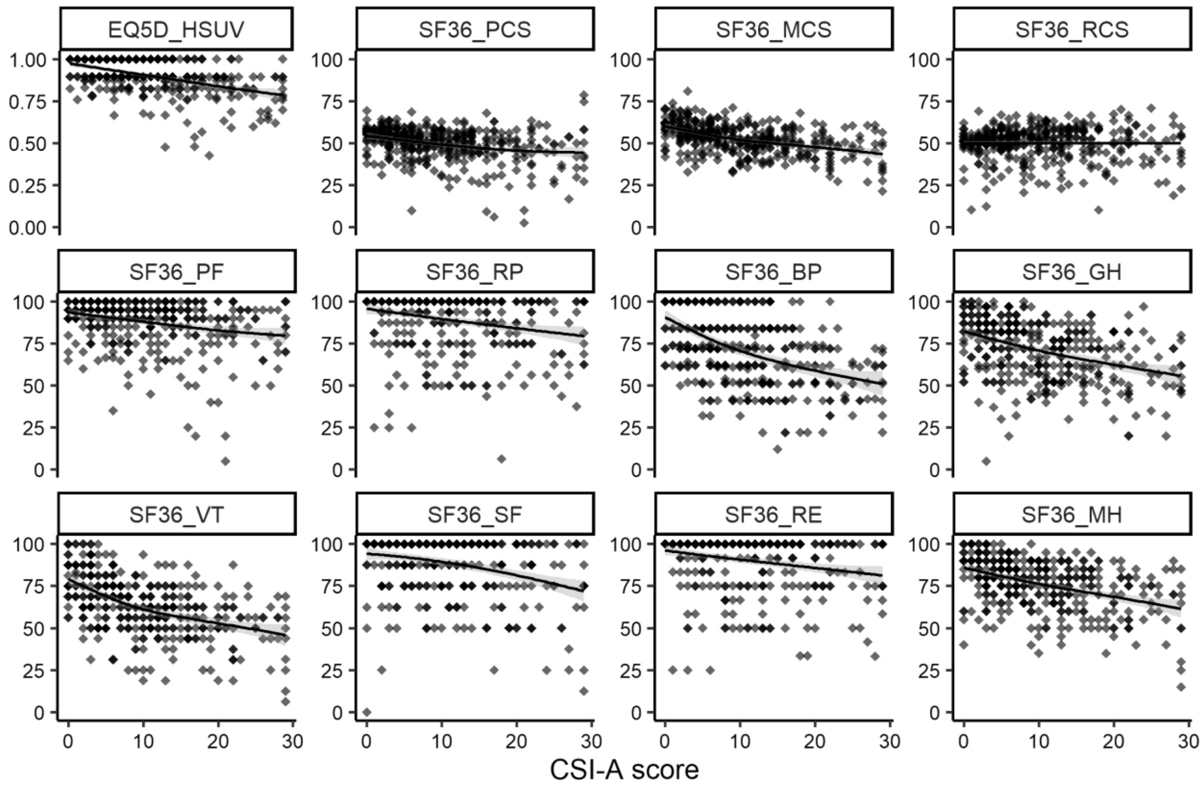

Supplemental Figure 2.
Correlation between CSI-A score and HRQOL indicators in SCI-A score <30 cohort

The spline model is based on the generalized additive model.

CSI-A, Central Sensitization Inventory, Part A; HRQOL, health-related quality of life; EQ5D, EuroQol 5 dimensions 5-level; HSUV, health state utility value; SF-36, 36-Item Short-Form Health Survey; PCS, physical component summary; MCS, mental component summary; RCS, role component summary PF, physical functioning; RP, role physical; BP, bodily pain; GH, general health; VT, vitality; SF, social functioning; RE, role emotional; MH, mental health.
