# Supplementary material for: Association between the central sensitization inventory score and health-related quality of life in community-dwelling middle-aged and older adults
Source: PLoS One. 2025 Oct 30;20(10):e0335923. doi: 10.1371/journal.pone.0335923 (PMC12574846; doi:10.1371/journal.pone.0335923)
Supplement: S1 Table — CSI-A, Central Sensitization Inventory, Part A; HRQOL, health-related quality of life; CI, confidence interval; EQ5D, EuroQol 5 dimensions; HSUV, health-state utility value; SF-36, 36-Item Short-Form Health Survey; PCS, physical component summary; MCS, mental component summary; RCS, role component summary PF, physical functioning; RP, role physical; BP, bodily pain; GH, general health; VT, vitality; SF, social functioning; RE, role emotional; MH, mental health. (PDF) [file pone.0335923.s003.pdf]

Supplemental Table 1.
Correlation between CSI-A score and HRQOL indicators in SCI-A score <30 cohort

	**Estimate**	**95% CI**	***P* value**
EQ5D HSUV	−0.492	−0.563, −0.412	<0.001
SF36 PCS	−0.296	−0.384, −0.202	<0.001
SF36 MCS	−0.474	−0.548, −0.393	<0.001
SF36 RCS	−0.041	−0.140, 0.059	0.42
SF36 PF	−0.279	−0.368, −0.184	<0.001
SF36 RP	−0.269	−0.359, −0.174	<0.001
SF36 BP	−0.506	−0.577, −0.428	<0.001
SF36 GH	−0.411	−0.490, −0.325	<0.001
SF36 VT	−0.508	−0.579, −0.431	<0.001
SF36 SF	−0.313	−0.400, −0.220	<0.001
SF36 RE	−0.249	−0.341, −0.154	<0.001
SF36 MH	−0.435	−0.512, −0.350	<0.001

CSI-A, Central Sensitization Inventory, Part A; HRQOL, health-related quality of life; CI, confidence interval; EQ5D, EuroQol 5 dimensions; HSUV, health-state utility value; SF-36, 36-Item Short-Form Health Survey; PCS, physical component summary; MCS, mental component summary; RCS, role component summary PF, physical functioning; RP, role physical; BP, bodily pain; GH, general health; VT, vitality; SF, social functioning; RE, role emotional; MH, mental health.
