# Supplementary material for: Association between the central sensitization inventory score and health-related quality of life in community-dwelling middle-aged and older adults
Source: PLoS One. 2025 Oct 30;20(10):e0335923. doi: 10.1371/journal.pone.0335923 (PMC12574846; doi:10.1371/journal.pone.0335923)
Supplement: S2 Table — CSI, Central Sensitization Inventory; HRQOL, health-related quality of life; CI, confidence interval; EQ5D, EuroQol 5 dimensions; HSUV, health-state utility value; SF-36, 36-Item Short-Form Health Survey; PCS, physical component summary; MCS, mental component summary; RCS, role component summary PF, physical functioning; RP, role physical; BP, bodily pain; GH, general health; VT, vitality; SF, social functioning; RE, role emotional; MH, mental health. *The fully adjusted model includes age, sex, comorbidities (hypertension, malignant neoplasm, diabetes, chronic kidney disease, angina or myocardial infarction, and cerebral stroke), and exercise habit as covariates. (PDF) [file pone.0335923.s004.pdf]

**Supplemental Table 2.**
**Multivariable linear regression analysis to determine the association of CSI-A score with HRQOL indicators in SCI-A score <30 cohort**

| | Unadjusted | | | Age- and sex-adjusted | | | Fully adjusted* | | |
|---|---|---|---|---|---|---|---|---|---|
| | **Beta** | **95% CI** | ***P* value** | **Beta** | **95% CI** | ***P* value** | **Beta** | **95% CI** | ***P* value** |
| EQ5D HSUV | −0.01 | −0.01, −0.01 | <0.001 | −0.01 | −0.01, −0.01 | <0.001 | −0.01 | −0.01, −0.01 | <0.001 |
| SF36 PCS | −0.39 | −0.52, −0.27 | <0.001 | −0.45 | −0.58, −0.33 | <0.001 | −0.42 | −0.54, −0.30 | <0.001 |
| SF36 MCS | −0.57 | −0.67, −0.46 | <0.001 | −0.56 | −0.66, −0.45 | <0.001 | −0.55 | −0.66, −0.45 | <0.001 |
| SF36 RCS | −0.05 | −0.18, 0.08 | 0.4 | −0.07 | −0.20, 0.06 | 0.3 | −0.07 | −0.20, 0.06 | 0.3 |
| SF36 PF | −0.53 | −0.71, −0.35 | <0.001 | −0.60 | −0.77, −0.42 | <0.001 | −0.56 | −0.73, −0.39 | <0.001 |
| SF36 RP | −0.60 | −0.81, −0.38 | <0.001 | −0.68 | −0.89, −0.48 | <0.001 | −0.64 | −0.85, −0.43 | <0.001 |
| SF36 BP | −1.4 | −1.7, −1.2 | <0.001 | −1.5 | −1.7, −1.2 | <0.001 | −1.4 | −1.7, −1.2 | <0.001 |
| SF36 GH | −0.95 | −1.2, −0.74 | <0.001 | −1.0 | −1.2, −0.81 | <0.001 | −0.98 | −1.2, −0.77 | <0.001 |
| SF36 VT | −1.2 | −1.4, −0.97 | <0.001 | −1.1 | −1.3, −0.95 | <0.001 | −1.1 | −1.3, −0.94 | <0.001 |
| SF36 SF | −0.75 | −0.98, −0.52 | <0.001 | −0.74 | −0.97, −0.51 | <0.001 | −0.72 | −0.95, −0.48 | <0.001 |
| SF36 RE | −0.54 | −0.75, −0.33 | <0.001 | −0.63 | −0.83, −0.42 | <0.001 | −0.62 | −0.83, −0.42 | <0.001 |
| SF36 MH | −0.86 | −1.0, −0.68 | <0.001 | −0.88 | −1.1, −0.70 | <0.001 | −0.87 | −1.1, −0.68 | <0.001 |

*The fully adjusted model includes age, sex, comorbidities (hypertension, malignant neoplasm, diabetes, chronic kidney disease, angina or myocardial infarction, and cerebral stroke), and exercise habit as covariates.

CSI-A, Central Sensitization Inventory, Part A; HRQOL, health-related quality of life; CI, confidence interval; EQ5D, EuroQol 5 dimensions; HSUV, health-state utility value; SF-36, 36-Item Short-Form Health Survey; PCS, physical component summary; MCS, mental component summary; RCS, role component summary PF, physical functioning; RP, role physical; BP, bodily pain; GH, general health; VT, vitality; SF, social functioning; RE, role emotional; MH, mental health.
